# Validity and Predictive Performance of Mini Nutritional Assessment Tool for Institutionalized Elders in Ethiopia

**DOI:** 10.1155/2022/6764657

**Published:** 2022-04-11

**Authors:** Abdu Oumer, Tariku Abebe, Kalkidan Hassan, Aragaw Hamza

**Affiliations:** ^1^Department of Public Health, College of Medicine and Health Sciences, Dire Dawa University, Dire Dawa, Ethiopia; ^2^Department of Nutrition and Dietetics, School of Public Health, College of Medicine and Health Sciences, Jimma University, Jimma, Ethiopia; ^3^Department of Anesthesia, College of Medicine and Health Sciences, Dire Dawa University, Dire Dawa, Ethiopia

## Abstract

**Background:**

Despite the high burden of malnutrition in the country, there is a lack of a simple and valid tool to screen elders in Ethiopia. The Mini Nutritional Assessment (MNA) tool has been validated for comprehensive geriatric assessment to identify malnutrition in other countries. However, there is a lack of evidence on the potential validity and reliability of the tool for institutionalized elders in Ethiopia. This study was aimed at determining the validity and predictive performance of MNA tool for malnutrition among Ethiopian institutionalized elderly.

**Methods:**

A facility-based survey was conducted on randomly selected 164 elders in geriatric centers to evaluate the validity, reliability, and predictive performance of full MNA against hemoglobin (Hgb) and ideal body weight (IBW) measured under standard procedures. The data was presented in ROC graphs, and reliability was evaluated with Cronbach alpha. The receiver-operating characteristic curve (ROC) analysis was used to assess the predictive performance of the tool. The area under the curve (AUC) with its 95% CI was reported. The Youden index, at maximum sensitivity and specificity, was used to obtain optimal cutoff points.

**Results:**

The internal consistency of the tool was good (*α* = 0.80). The full MNA score can better predict Hgb (AUC = 0.845; 0.783-0.899) and percentage of IBW (AUC = 0.90; 0.842-0.941) at specified cutoff points. A full MNA can predict malnutrition or risk of malnutrition based on percentage IBW at a sensitivity and specificity of 97.3% and 72.2%, respectively.

**Conclusions:**

The full MNA has the potential to be a reliable and valid nutritional assessment tool for institutional elders.

## 1. Introduction

Due to access to health care and epidemiologic transitions, the current population age structure is changing, with an increasing aged population. In sub-Saharan Africa, the estimated old age segment of the population will reach above 34 million by 2030 [1]. In Ethiopia, an estimated 8.1 million of the population is estimated to be over 65 years old with an increasing trend [2]. However, this increasing life expectancy needs comprehensive care for a better quality of care. Nutrition is a key aspect and determinant of geriatric health, and nutritional assessments are an important aspect of comprehensive geriatric evaluation [3, 4]. Timely screening allows detecting malnutrition and the risk of malnutrition early which enables to intervene early before complications occur [3].

The Mini Nutritional Assessment (MNA) is one of the most widely used and studied nutrition screening/assessment tools [5]. It is a widely used nutritional screening tool that has been validated in other countries in different settings. It has eighteen items covering four dimensions (anthropometry, dietary assessment, global assessment, and self-evaluation) [6]. As compared to other screening tools, the MNA is appropriate and can potentially provide a proxy estimate of the nutritional status of the elderly [7]. It has comprehensive geriatric assessment criteria, including health, nutrition, psychological, and social aspects [8]. The Full MNA contains 18 questions to assess nutritional status using subjective assessment, global assessment, and anthropometric data, scored out of 30. Thus, elderly people with MNA scores of below 17 and 17 to 23.5 are classified as malnourished and at risk of malnutrition, respectively [9].

The recent rapid rise in the elderly population will undoubtedly pose several challenges. The implications of aging are multiple and multifaceted, and most of the time they go unrecognized. As age advances, there will be a steady decline in physical and biological functions. The magnitude of malnutrition in the elderly is underreported [10], which needs a validated simple screening tool for better identification and management of cases.

So far, the MNA tool has been tested for validity and reliability in different research and settings [11–13]. The MNA tool is validated and checked for reliability in Ethiopian community dwellers elderly [14], which focused on community dwellers and used body mass index, which may not be appropriate [15, 16]. As a result, there is a need to validate the tool for institutionalized elderly in Ethiopia using gold standard biochemical indicators (hemoglobin) and percent of ideal body weight, where no such evidence exists for or against it. Therefore, the present study was conducted to test the validity and reliability of the full MNA screening tool among the elderly population living in a geriatric center in Ethiopia.

## 2. Methods

### 2.1. Study settings and design

The study area is located approximately 515 km from the capital city of Ethiopia, Addis Ababa. Dire Dawa covers an area of 530 square kilometers and is found at a latitude and longitude of 9°36′ 0^″^N 41°′52^″^E with an elevation of 1204 meters above sea level. Based on the 2019/2020 population projection by the Central Statistical Agency of Ethiopia, Dire Dawa administration has a total population of 506,609 (248,238 males and 258,371 females). Thus, the current number of elderly people in the administration is estimated to be about 16,700 [17]. The administration has nine urban and 38 rural kebeles (“kebeles” refers to the fourth-level administrative unit), and there are also six hospitals. There are two geriatric centers with a total of 350 elderly people [18]. A facility-based survey was conducted among randomly selected institutionalized elderly people. The study excluded elderly people with severe edema, severe vertebral deformity, or who were critically ill without a caregiver.

### 2.2. Sample size determination

The optimum sample size was estimated for the sensitivity, specificity, and area under the ROC curve separately. The Buderer's formula was used for sample size calculation in diagnostic accuracy studies at the required absolute precision level for sensitivity and specificity [19]. Using the sensitivity (95%) (equation (1)) and the prevalence of malnutrition estimate (50.9%) [20], at a 95% confidence level, from the previous study, the sample size became 143. Similarly, for specificity (95%) of the MNA tool (equations ((1)) and ((2))) and the prevalence estimate (50.9%) [20], the final sample size became 149.

(1) $$\begin{array}{c} \frac{Z^{2}\alpha {/}_{2}\text{SN}\left(1-\text{SN}\right)}{\epsilon^{2}\;X\text{ Prev}}, \end{array}$$

(2) $$\begin{array}{c} \frac{Z^{2}\alpha {/}_{2}\text{Sp}\left(1-\text{Sp}\right)}{\epsilon^{2}X\left(1-\text{Prev}\right)}, \end{array}$$

where *Zα*/_2_ is a standard normal value at *α* level of 5% and 95% confidence level, SN is anticipated sensitivity (95%), SP is anticipated specificity (95%), Prev is prevalence of malnutrition among older adults (50.9%) [20], and *ϵ* is required absolute precision on either side of the sensitivity (0.05%). Based on the sensitivity and specificity, the minimum sample size required becomes 149.

Finally, the sample size for estimating the diagnostic accuracy of the MNA tool was estimated using the detailed calculation as stated in [19]. Using the binomial-based variance of the area under the curve (AUC) and equal sample size (*n*1 = *n*2), the sample was estimated using equation (3) [21].

(3) $$\begin{array}{c} n=\frac{Z^{2}\alpha {/}_{2}V\left(\text{AUC}\right)}{\epsilon^{2}}. \end{array}$$

- **Zα**/_2_ is a standard normal value at **α** level of 5% (95% confidence level)
- **ϵ** is the required absolute precision on either side of the AUC (0.07)
- **n** is the minimum sample required, and **V**(**AUC**) is the variance of the AUC calculated based on the formula
- *V*(AUC) = (0.0099 *X* *e*^−*ɑ*/2^) × (6*ɑ* + 16)
- *ɑ* = *ϕ*^−1^(AUC) *X* 1.414, and *ϕ*^−1^ is the inverse of standard cumulative normal distribution
- *ɑ* = *ϕ*^−1^(0.70) *X* 1.414 = 0.741502
- *V*(AUC) = (0.0099 *X* *e*^−0.741502^) × (6 × 0.741502^2^ + 16) = 0.145136

Substituting the above empirical evidences to equation (3), the estimated sample size for properly identifying the diagnostic accuracy of the test became 114. Taking the larger sample size with the addition of the nonresponse rate, a total of 164 elders were required. The total sample size was proportionally allocated to each geriatric center, and individuals were selected using a lottery method.

### 2.3. Methods of Data Collection

A pretested structured questionnaire including the full MNA tool adapted from Nestle and other important variables was used to collect data. The tool was prepared in English and translated into three local languages consistently, and the data was collected by a trained nurse, laboratory technician, and supervisors. All mandatory COVID-19 prevention precautions were maintained. In addition, the weight, height, and midupper arm circumference (MUAC) were measured under standard procedures. A point-of-care Hgb meter using HemoCue machine (HemoCue analyzer 301, Angelholm, Sweden) was used to determine the Hgb level under standard laboratory procedures. This method uses a small blood sample, with little skills and higher feasibility in field settings [22–24]. Studies showed that HemoCue analyzer 301 seems to be accurate enough for diagnosing anemia at population level. However, there is a concern that point-of-care Hgb testing might be suboptimal due to problems in testing procedures [25]. To reduce the potential bias significantly, we trained the data collectors on the standard laboratory procedures and a quality checked materials and stripes were applied.

### 2.4. Study Variables

The outcome variable of this study is the MNA score, while demographic characteristics (age, sex, educational status, marital status, and type of primary caregiver), alcohol drinking, hemoglobin level, anthropometric variables (body mass index, percent of ideal body weight, and midupper arm circumference), and other important variables were considered independent variables.

In this study, undernutrition is defined as a composite indicator of malnutrition (MNA score < 17 points) and at risk of malnutrition (MNA score from 17 to 23.5) based on the full MNA score cutoff point [26]. Anemia was defined as a hemoglobin level for male < 13 g/dl and for female < 12 g/dl. A percentage of IBW < 70%, 70–79%, and 80-89% denote severe, moderate, and mild malnutrition.

### 2.5. Data Processing and Analysis

SPSS version 25.0 and MedCalc V. 19 were used to analyze the data. The information was summarized and described using frequency, means, standard deviations, tables, graphs, and other methods. In addition, the ideal body weight (IBW), which is the target healthy body weight [27], was estimated based on the Hamwi's equation for males and females, separately, considering the frame skeleton size, as explained elsewhere [28, 29]. Then, the percent of IBW was calculated as a percentage of actual weight.

As a measure of the reliability of the MNA, homogeneity was computed using Cronbach's *α*. A Cronbach's *α* value of 0.60, 0.70, and 0.80 was considered acceptable, adequate, and good, respectively [30]. The internal consistency of the MNA tool was evaluated by Spearman's rank correlation coefficients [31]. Sensitivity, specificity, positive predictive value, and negative predictive value were estimated.

We used a receiver-operating characteristic (ROC) analysis to identify optimal threshold values for predicting malnutrition and evaluate predictive performance of the MNA tool against anemia or percentage of IBW for screening malnutrition among elderly. It allows to fit a model for evaluating the diagnostic accuracy of a particular tool against a relatively valid tool. The area under the curve (AUC) reported with a value of 1.0 indicates perfect discrimination [32]. AUC with 95% CI was reported. Points at maximum Youden index (sensitivity + specificity − 1) were used to identify the best MNA cutoff point for the identification of malnutrition [33]. *P* value below 5% was used to declare statistical significance level.

### 2.6. Data Quality Assurances

The tool was prepared in English and translated into local languages by English-language professionals. Before actual data collection, training was given to data collectors and supervisors on the study procedure, tools, and how to collect data. Extensive hands-on training was given for weight, height, calf circumference, MUAC, and other anthropometric measurements. The Hgb measurement was done using a valid and reliable HemoCue machine under standard operating procedures. A pretest was conducted on 5% of the sample outside of the sampling population. Anthropometric reliability assessment using technical error of measurement (TEM) was calculated for each anthropometric measurement. The intra and interobserver TEM were calculated to pick reliable measurers. Questionnaire completeness, accuracy, and consistency were checked on a daily basis.

### 2.7. Ethical Approval

Ethical approval was obtained from Dire Dawa University's Institutional Ethical Review committee. A written informed consent was obtained from each participant or their care giver if applicable. Confidentiality was kept, and the information will not be shared.

## 3. Results

### 3.1. Sociodemographic and Lifestyle Characteristics

In this study, 164 elderly people participated in the study where 81 (49.4%) were men. The age of respondents ranges from 65 to 92 years and mean age of 74.6 (±7.9 SD) years. Among the study participants, 93 (56.7%) were unable to read and write, while 80 (48.8%) of study subjects were widowed with their partner died (Table 1).

Regarding lifestyle characteristics, 19 (11.6%) smoked cigarettes. Compared to women (5.3%), higher proportion of men 9.47% smoked cigarettes. In addition, 35 (21.3%) of the elderly drink alcohol. Among the majority of men, 33 (94.3%) were involved in alcohol drinking more than women (25.7%).

### 3.2. Nutritional Status of Elderly

Based on the malnutrition screening, 40.9% (33.8 to 48.4%) and 40.2% (32.7 to 47.7%) of the elderly were well-nourished and malnourished, respectively, while 18.9% (3.30 to 24.9%) of the elderly (scored between 17 and 23.5) were at risk of malnutrition. The prevalence of malnutrition and the risk of malnutrition among men was 55.3%, which was higher than among women (40.2%) (Table 1). Based on a percentage of IBW, 31.7% were malnourished as compared to 59.2% based on Hgb cutoff point criteria, while 37 (44.6%) and 46 (55.4%) of males and women were anemic, respectively.

### 3.3. Reliability of the Full MNA Tool

The internal consistency of the MNA tool as measured by Cronbach's coefficient was found to be 0.797. Omitting calf circumference from MNA items significantly lowered the Cronbach's value, and the reliability of the tool became 0.772, 0.773, and 0.777. This shows that CC is the most important item in the tool to assess the nutritional status of the elderly. Conversely, deleting live independently, the value of Cronbach's increased to 0.801. Therefore, living independently has contributed to increasing the overall reliability of the MNA tool in this study (Table 2).

Overall, there was a significant positive correlation between the total MNA score and the eighteen items of the MNA tool, which strengthens the reliability of the MNA tool. It is indicated that CC and BMI showed a significant positive correlation to the total MNA score (*r* = 0.766, *P* < 0.001 and *r* = 0.693, *P* < 0.001). Live independently and pressure sores, protein intake, and more than three prescription drugs showed poor correlation to the total MNA score (*r* = 0.199, *P* < 0.001 and *r* = 0.218, *P* < 0.005, respectively). As a result, poor correlation of these four parameters was observed, and they all showed a higher Cronbach's *α* if they were omitted from the tool (Table 2).

Correlating the total MNA score to a percentile of IBW and hemoglobin measurements as criteria, a significant positive correlation was observed between the total MNA score, hemoglobin, and percentage of IBW. Among these, MNA score showed a higher positive correlation to the percentage of the IBW (*r* = 0.70, *P* < 0.001) and a lower correlation against Hgb (*r* = 0.43, *P* < 0.001), respectively. Concurrent validity of the instrument showed the correlation between total MNA scores and the patients' view of their own nutritional status of participants. There was a positive correlation which resulted in *r* = 0.604 (*P* < 0.001) (Table 2).

### 3.4. Validity and Predictive Performance of the MNA Tool

**Figure 1:**
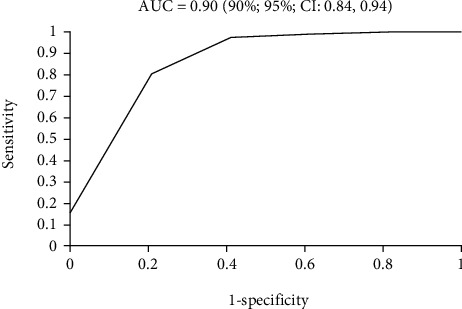
Predictive performance (ROC) of the MNA tool for Hg hemoglobin among elders in Ethiopia.

**Figure 2:**
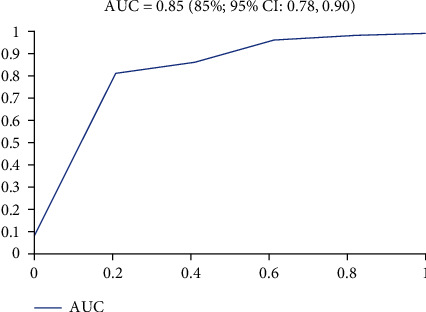
Predictive performance (ROC) of the MNA tool for percent of ideal body weight among elders in Ethiopia.

The MNA tool was tested against hemoglobin and the percentile of IBW, for their predictive performance. The predictive ability of the full MNA score for Hgb (less than 13 for male and l12 for female) and percent of IBW cutoff point (less than 90%) was 0.85 (AUC = 0.85; 95% CI 0.78, 0.90, and *P* < 0.0001) and 89.9 (95% CI: 0.842, 0.941, *P* < 0.0001, respectively) (Table 3). This indicates that the MNA test is relatively accurate in detecting malnourished people according to hemoglobin and percentage IBW (Figures 1 and 2).

The sensitivity, specificity, positive predictive value, and negative predictive value of the full MNA according to its established cutoff points for predicting the percentile of IBW were 97.3%, 72.2%, 73.6%, and 87.0%, respectively. Using, the Youden index, the best cutoff point to detect malnourished and at risk of malnutrition in the present study was ≤23.5, which resulted in similar sensitivity, specificity, positive predictive value, and negative predictive value.

According to its established cutoff points for predicting hemoglobin (female 12 g/dl and male 13 g/dl), the MNA's sensitivity, specificity, positive predictive value, and negative predictive value were 87.36%, 72.73%, 78.4%, and 84%, respectively. Using the Youden index, the best cutoff point to detect malnourished and at risk of malnutrition in the present study was ≤19.5, resulting in a considerable decrease in sensitivity and positive predictive value of 81.6% by 5.76% and an increase in specificity and negative predictive value by 8.87%, respectively (Table 3).

## 4. Discussions

This study was conducted with the objective of evaluating the MNA tool to identify malnutrition and risk for malnutrition among the elderly population in a geriatric center. The majority, 40.9% (95% CI: 33.8%-48.42%) and 40.2% (95% CI: 32.7%–47.7%), were found to be well nourished and malnourished, while 18.9% (95% CI: 13.30%–24.89%) were at risk of malnutrition according to full MNA score. Based on a percentage of IBW and Hgb cutoff points, 31.7% and 59.2% were malnourished.

The reliability of the tool was checked using Cronbach's *α* coefficient (0.80), indicating acceptable consistency. This showed better reliability as compared to studies in Hawassa and Iran, which had a 0.65 and 0.6, respectively [9, 14]. The full MNA tool can be reliably used for nutritional screening, which allows one to identify malnutrition in resource poor settings. In addition, there is a significant correlation between the items of the MNA tool and the MNA score, indicating that items measure the outcome in a similar way. Study from Brazil on institutionalized elderly also showed that the tool is reliable in identifying malnutrition. A study from Ethiopia and Brazil study showed pressure sores and multiple drug prescriptions had a significant correlation attributable to being in institutions and care [34, 35]. The MNA tool can be used reliably to screen the elderly people for identifying malnutrition and risk of malnutrition.

The validity of the MNA tool was validated against percent of IBW (*r* = 0.70) and Hgb (*r* = 0.43), which are valid and reliable assessments of nutritional status. This finding suggests that the tool was valid and can identify the nutritional status of an elderly population with better predictive ability for IBW and Hgb. The MNA score can predict low Hgb (AUC = 0.85), which makes the MNA tool to have a better predictive performance. Similarly, study from Ethiopia showed that the MNA can predict malnutrition based on BMI (AUC = 0.85) [14]. These findings showed the MNA tool was valid and accurate to assess the nutritional status of elderly people in Ethiopia.

In addition, the MNA has a better predictive performance for percent of IBW (AUC = 0.90), which shows excellent predictive performance or agreement. Other studies also showed a better predictive power for malnutrition among elders (AUC = 0.91) [35].

The performance of the tool for predicting IBW at an established cutoff point was great (sensitivity, specificity, positive predictive value, and negative predictive value of 97.3%, 72.2%, 73.56%, and 87%, respectively). Validations at the start also reported a higher sensitivity (96%) and specificity (98%) than the current study [36] and consistent with the trial in Nepal and Ethiopia [14]. Similarly, a better prediction was seen for Hgb, which is consistent with the results from Ethiopia and Iran [9, 14]. The current study's findings on sensitivity and specificity were similar, but lower specificity with the study revealed from Iran, which was 82% and 88%, respectively [27].

The MNA score ≤ 19.5 and those less than 23.5 can identify malnutrition and those at risk of malnutrition among the elderly in Ethiopia. This cutoff point for Hgb is slightly higher than nestle cutoff point (17), while the cutoff point for IBW is almost similar to nestle ranking. Considering the rigorous analysis and the validity of the tool so far, the MNA can be used to screen elderly people for identifying risk and established malnutrition with the current cutoff point [37].

A few studies were conducted based on the validity of MNA tool in Ethiopia. It is easily administered by peripheral or primary level health staff, without the need for specific training in nutrition. In Ethiopia, primary level health personnel, with little skills, are responsible for clinical care, where MNA can help them easily assess the nutritional status of the elderly in resource limited settings.

This study is first of its kind to pinpoint and assess the validity and reliability of the MNA tool using valid and reliable nutritional assessment methods (Hgb and % IBW), which allows to pick the outcome more accurately. However, the results of this study should be thought in the light of smaller sample size and the survey nature of the data that may affect the result.

## 5. Limitations of the Study

The findings of this study shall be thought considering some limitations of the study. Anthropometric measurements in older adults might introduce some slight measurement bias. The point-of-care Hgb testing may not be hundred percent valid and might under or overestimate the Hgb measurement compared to laboratory testing. The small number of the target population (*n* = 164) and being among institutionalized older adults might limit the generalizability of the result.

## 6. Conclusion

The full MNA tool is a reliable and valid nutrition assessment method for identifying malnutrition and those at risk of malnutrition among the institutionalized elderly in Ethiopia. The Nestle established cutoff point for at risk and established malnutrition can predict nutritional status and applied in Ethiopia.

## Figures and Tables

**Table 1:** Sociodemographic characteristics and nutritional status by sociodemographic and life style characteristics among institutionalized elders in eastern Ethiopia.

		Malnutrition status classification by MNA (frequency and percentage in bracket)					
		Malnourished		At risk of malnutrition		Well nourished	
		Male	Female	Male	Female	Male	Female
Age group	65-69	11 (28.9)	8 (28.6)	0 (0)	7 (43.8)	10 (35.7)	14 (35.9)
	70-74	5 (42.1)	4 (14.3)	5 (33.3)	0 (0)	6 (21.4)	10 (25.6)
	75-79	11 (28.9)	5 (17.9)	3 (20)	4 (25)	4 (14.3)	0 (0)
	80 and above	11 (28.9)	11 (39.3)	7 (46.7)	5 (31.3)	8 (28.6)	15 (38.5)

Educational status	Illiterate	17 (44.7)	22 (78.6)	3 (20)	9 (56.3)	9 (32.1)	33 (84.6)
	Read and write	12 (31.6)	5 (17.9)	4 (26.7)	3 (18.8)	11 (39.3)	3 (7.7)
	Grades 1-8	7 (18.4)	1 (3.6)	4 (26.7)	4 (25)	5 (17.9)	3 (7.7)
	Grade 9 and above	2 (5.3)	0 (0)	4 (26.7)	0 (0)	3 (10.7)	0 (0)

What is your marital status	Married	10 (26.3)	7 (25)	5 (33.3)	1 (6.3)	7 (25)	6 (15.4)
	Widowed	19 (50)	19 (67.9)	6 (40)	11 (68.8)	6 (21.4)	19 (48.7)
	Single	5 (13.2)	2 (7.1)	2 (13.3)	2 (12.5)	9 (32.1)	8 (20.5)
	Divorced	4 (10.5)	0 (0)	2 (13.3)	2 (12.5)	6 (21.5)	6 (15.4)

Type of caregiver	Partner	3 (7.9)	1 (3.6)	0 (0)	1 (6.3)	1 (3.6)	0 (0)
	Child	6 (15.8)	4 (14.3)	2 (13.3)	3 (18.8)	4 (14.3)	7 (17.9)
	Relatives	4 (10.5)	0 (0)	0 (0)	0 (0)	3 (10.7)	1 (2.6)
	Nurse	23 (60.5)	23 (82.1)	12 (80)	11 (68.8)	19 (67.9)	31 (79.5)
	No one	2 (5.3)	0 (0)	1 (6.7)	1 (6.3)	1 (3.6)	0 (0)

Smoke cigarette	Yes	8 (21.1)	0 (0)	4 (26.7)	1 (6.3)	6 (21.4)	0 (0)
	No	30 (78.9)	28 (100)	11 (73.3)	15 (93.8)	22 (78.6)	39 (100)

Drink alcohol	Yes	11 (28.9)	1 (3.6)	10 (66.7)	1 (6.3)	12 (42.9)	0 (0)
	No	27 (71.1)	27 (96.4)	5 (33.3)	15 (93.8)	16 (57.1)	39 (100)

**Table 2:** Correlation between total MNA score and eighteen items of MNA among institutionalized elders in eastern Ethiopia.

Item no.	MNA constructs	Coefficients (*r*)	*P* value	Cronbach's alpha if item deleted
1	Change dietary intake	0.625	<0.001^∗∗^	0.773
2	Weight loss	0.497	<0.001^∗∗^	0.790
3	Mobility	0.359	<0.001^∗∗^	0.792
4	Psychological stress or acute disease	0.525	<0.001^∗∗^	0.787
5	Neuropsychological problems	0.450	<0.001^∗∗^	0.789
6	BMI	0.693	<0.001^∗∗^	0.791
7	Live independently	0.199	<0.001^∗^	0.801
8	More than three prescription drugs per day	0.237	<0.002^∗^	0.797
9	Pressure sores or skin ulcers	0.218	<0.005^∗^	0.798
10	Full meals do you eat daily	0.574	<0.001^∗∗^	0.777
11	Protein intake	0.160	<0.001^∗^	0.797
12	Fruits and vegetables	0.250	<0.001^∗∗^	0.795
13	Fluid consumed per day	0.444	<0.001^∗∗^	0.791
14	Mode of feeding	0.524	<0.001^∗∗^	0.784
15	Self-view of nutritional status	0.604	<0.001^∗∗^	0.778
16	Perceived health status	0.544	<0.001^∗∗^	0.780
17	MUAC	0.652	<0.001^∗∗^	0.785
18	Calf circumference	0.766	<0.001^∗∗^	0.772

BMI: body mass index; MNA: Mini Nutritional Assessment; MUAC: midupper arm circumference; *r*: Pearson correlation, ^∗^Significant at *P* < 0.05.

**Table 3:** Accuracy of the full MNA tool with respect to Hgb and percentile of IBW among institutionalized elders in eastern Ethiopia.

Measures	Sensitivity (95% CI)	Specificity (95% CI)	+PPV (95% CI)	-PPV (95% CI)	AUC
Hg	81.6 (71.9-89.1)	79.2 (68.5-87.6)	81.6 (73.9-87.4)	79.2 (70.7-85.8)	Acceptable
% IBW	97.3 (90.6-99.7)	72.2 (61.8-81.1)	73,6 (65.9-80.0)	87,0 (78.8-92.4)	Acceptable

## Data Availability

All relevant data are within the manuscript.

## Abbreviations

AUC: Area under the curve
BMI: Body mass index
CC: Calf circumference
MNA: Mini Nutritional Assessment tool
MUAC: Midupper arm circumference
IBW: Ideal body weight
ROC: Receiver-operating characteristics
TEM: Technical error of measurement
WHO: World Health Organization.
